# Acceptability – a neglected dimension of access to health care: findings from a study on childhood convulsions in rural Tanzania

**DOI:** 10.1186/1472-6963-12-113

**Published:** 2012-05-09

**Authors:** Angel Dillip, Sandra Alba, Christopher Mshana, Manuel W Hetzel, Christian Lengeler, Iddy Mayumana, Alexander Schulze, Hassan Mshinda, Mitchell G Weiss, Brigit Obrist

**Affiliations:** 1 Ifakara Health Institute, Off Mlabani Passage, P.O.Box 53,, Ifakara,, Morogoro, Tanzania; 2 Swiss Tropical and Public Health Institute, Socinstrasse 57, CH-4002, Basel, Switzerland; 3 University of Basel, Basel, Switzerland; 4 Papua New Guinea Institute of Medical Research, Goroka, EHP 441, Papua New Guinea; 5 Novartis Foundation for Sustainable Development, WRO-1002.11.56, CH-4002, Basel, Switzerland; 6 Tanzania Commission for Science and Technology, P.O. Box 4302,, Dar es Salaam, Tanzania; 7 Institute of ethnology, University of Basel, Petersplatz,, Basel, Switzerland; 8 The University of Queensland, School of Population Health, Herston, Qld, 4006, Australia

## Abstract

**Background:**

Acceptability is a poorly conceptualized dimension of access to health care. Using a study on childhood convulsion in rural Tanzania, we examined social acceptability from a user perspective. The study design is based on the premise that a match between health providers’ and clients’ understanding of disease is an important dimension of social acceptability, especially in trans-cultural communication, for example if childhood convulsions are not linked with malaria and local treatment practices are mostly preferred. The study was linked to health interventions with the objective of bridging the gap between local and biomedical understanding of convulsions.

**Methods:**

The study combined classical ethnography with the cultural epidemiology approach using EMIC (Explanatory Model Interview Catalogue) tool. EMIC interviews were conducted in a 2007/08 convulsion study (n = 88) and results were compared with those of an earlier 2004/06 convulsion study (n = 135). Earlier studies on convulsion in the area were also examined to explore longer-term changes in treatment practices.

**Results:**

The match between local and biomedical understanding of convulsions was already high in the 2004/06 study. Specific improvements were noted in form of (1) 46% point increase among those who reported use of mosquito nets to prevent convulsion (2) 13% point decrease among caregivers who associated convulsion with ‘evil eye and sorcery’, 3) 14% point increase in prompt use of health facility and 4)16% point decrease among those who did not use health facility at all. Such changes can be partly attributed to interventions which explicitly aimed at increasing the match between local and biomedical understanding of malaria. Caregivers, mostly mothers, did not seek advice on where to take an ill child. This indicates that treatment at health facility has become socially acceptable for severe febrile with convulsion.

**Conclusion:**

As an important dimension of access to health care ‘social acceptability’ seems relevant in studying illnesses that are perceived not to belong to the biomedical field, specifically in trans-cultural societies. Understanding the match between local and biomedical understanding of disease is fundamental to ensure acceptability of health care services, successful control and management of health problems. Our study noted some positive changes in community knowledge and management of convulsion episodes, changes which might be accredited to extensive health education campaigns in the study area. On the other hand it is difficult to make inference out of the findings as a result of small sample size involved. In return, it is clear that well ingrained traditional beliefs can be modified with communication campaigns, provided that this change resonates with the beneficiaries.

## Background

Acceptability is a neglected and poorly conceptualized dimension of access to health care. In earlier papers, we presented a new framework for the study of access to health care in contexts of livelihood insecurity [1], and examined three dimensions of access, namely availability, affordability and accessibility, in the case of fever and mild malaria episodes in rural Tanzania [2-4]. The present paper examines acceptability as a dimension of access, using convulsion episodes in under-five-year-old children in the same study area.

Drawing on the seminal paper of Penchansky and Thomas [5], we understand “access as a concept representing the degree of ‘fit’ between the clients and the system”. In this view, acceptability can be seen as “the relationship of clients’ attitudes about personal and practice characteristics of providers to the actual characteristics of existing providers, as well as to provider attitudes about acceptable personal characteristics of clients” [5]. The term acceptability was used to describe consumer reaction to personal characteristics of providers like sex or ethnicity, to the type of facility, to the religious affiliation of the facility or provider or the neighborhood of the facility. Providers, in turn, were described as having attitudes with regard to preferred attributes of clients or financing mechanisms, showing for instance unwillingness to serve welfare patients.

More recently, studies examine the acceptability of products, technologies and associated services or campaigns, for instance hormonal contraception methods [6] or the mass vaccination campaign against A/H1N1 2009 pandemic-influenza in France [7]. The term social acceptability is used in studies examining whether preventive measures or treatments are perceived as acceptable by the general population [8]. This study is however based on the idea that the concept of ‘acceptability’ has not been well defined and studied in the context of trans-cultural societies and with regard to what attributes constitute the concept.

Guided by perspectives developed in medical anthropology, we use the term social acceptability to emphasize that individual perceptions are influenced by social representations and modified in social interactions. As indicated in Penchansky and Thomas [5], we suggest that a ‘fit’ or match between providers and clients with regard to their understandings of disease is of particular relevance. The usefulness of such an approach is most obvious if striking differences exist in providers’ and clients’ views about the causes and treatment of health problems, as has been reported, for instance, for high fever and convulsions in Africa and Asia [9,10]. In areas of high malaria endemicity, health providers with biomedical training treat fever and convulsions with anti-malarials and antibiotics, while local communities traditionally considered convulsions as distinct illnesses which are more amenable to local treatment practices [9,11,12]. Recent studies in Tanzania have also indicated use of modern treatment for convulsion [13,14]. In Tanzania, the KiSwahili term *degedege* is used for convulsions in children and certain preventive and treatment practices were associated with this well-known illness category [15-17].

According to the current guidelines for malaria control and Integrated Management of Childhood illneses (IMCI) [18], children above one month of age with convulsions are treated with diazepam at clinics, while phenobarbitone is advised for young infants less than one month of age. In cases of severe febrile disease or severe malaria, intramuscular quinine, antibiotics and paracetamol have to be administered at clinics and follow up referral to a hospital is recommended.

Over the past decades, the Tanzanian health system, often supported by projects and programs, has made great efforts to control malaria. In the study area, three initiatives explicitly aimed at increasing the match between biomedical and local understanding and treatment of malaria: the KilomberoTreated Net (KINET) Project (1996–2000), which implemented a social marketing distribution system for insecticide treated nets to prevent malaria [19]; the training of health providers in Integrated Management of Childhood Illnesses (IMCI) introduced in 2002 which explicitly refers to *degedege,* its symptoms, causes and treatment; and, since 2004, the ACCESS program, which aims at improving access to prompt and effective treatment for malaria among children, pregnant mothers and the community at large [20].

The ACCESS program directs interventions to the community (i.e. the home), the drug shops and the health facilities. In the *community,* the ACCESS program conducts social marketing campaigns for prompt and effective treatment of mild and severe forms of malaria. The campaign activities include road shows with role plays, dancing and public lectures and the distribution of Information, Education and Communication (IEC) materials like posters, billboards, stickers, T-shirt and caps with malaria-related messages. Role plays address, local ideas, linking convulsions with spirits, the ‘*degedege* bird’ and evil eyes and promote a new understanding linking *degedege* with severe malaria. For example, an IEC message on a T-shirt reads, “Convulsion is a sign of severe malaria and can be treated at the health facility” (“*Degedege ni dalili ya malaria kali na inatibika vituo vya afya*”). Similarly, local counterproductive treatment practices were discouraged and alternatives were recommended, for instance a public health advises message: “Do not urinate on a convulsed child but take her to a health facility immediately” (“*Usimkojolee mtoto mwenye degedege ila mpeleke kituo cha afya mara moja”).* The importance of using Insecticide Treated Nets (ITNs) as a measure to prevent malaria has also been emphasized in the campaigns.

In *drug shops*, the ACCESS program collaborates with the Accredited Drug Dispensing Outlets (ADDO) program of the Government of Tanzania which is implemented by Management Sciences for Health (MSH) and the Tanzania Food and Drug Authority (TFDA). This program provides not only training for drug sellers but also assistance in the up-grading of the part II drug shops (allowed to sell over the counter medicine only) including mechanisms for proper storage of medicines.

In *health facilities,* the ACCESS program supports the Council Health Management Team in improving the supervision of health staff in the 75 dispensaries, 7 health centers and 4 hospitals of the Kilombero and Ulanga districts. In the course of their visits, the supervisory teams assess, for instance, whether health providers adhere to the national malaria guidelines and the IMCI guidelines in their day-to-day case management.

The study presented here has grown out of the monitoring and evaluation component of the ACCESS program. It explores changes in the social acceptability of biomedically recommended malaria treatment, paying particular attention to the match between providers’ and clients’ understanding of convulsions in children under the age of five. The assumption is that the understandings of the clients, or more precisely of the sick children’s caregivers, manifest themselves in their recognition of symptoms, interpretation of causes and health seeking behaviour. The better the reported symptoms, causes and treatment seeking correspond to the biomedical guidelines, the closer the match and the higher the social acceptability of the biomedically recommended treatment for severe malaria.

## Methods

### Study area

The study was conducted in the Kilombero Valley in south-eastern Tanzania, a rural area with villages lined up along the borders of a flood plain formed by the Kilombero River. Most villagers rely on agriculture for their livelihood and grow rice, maize and cassava as main crops. The main administrative and commercial centre is Ifakara, a bustling town with a population of about 59,497 in 2010 (pers. comm. Kilombero District Planning Officer). The malaria transmission was perennial and intense in the nineties [21], but there is evidence of a decline in recent years [22].

The study was confined to the twenty-five villages covered by the Kilombero and Ulanga Health Demographic Surveillance System (HDSS) with a total population of about 74,000 in 2004 and over 92,000 in 2008 [4]. The area is covered by 13 public and private health facilities (11 dispensaries and 2 health centres) with 55 and 135 ADDOs in Ulanga and Kilombero districts respectively [4].

### Study design

The study combined classic ethnography with cultural epidemiology as defined by Weiss [23,24]. The aim was to investigate the social acceptability of anti-malarials with a focus on the understanding of convulsions from the perspective of the caregivers of under-five-year-old children. To capture potential changes in the course of the ACCESS program, two rounds of interviews were carried out:1) from November 2004 to March 2006 [14] and 2) from September 2007 to November 2008. This paper compares the findings of both sub-studies and examines them with reference to the ACCESS framework [1]. Earlier studies on convulsion in the study area were also examined to explore changes in treatment seeking for convulsion.

In each sub-study, a semi-structured interview guide called Explanatory Model Interview Catalogue - EMIC [23,24] was administered to caretakers two to four weeks after a HDSS field worker had identified and reported the convulsion episode. The study followed the same sampling approach as for the previous study [14] where a total of 88 convulsion cases were continuously extracted from the HDSS records for the period of 15 months and followed up by trained ACCESS field staff who conducted the EMIC interviews in KiSwahili. They only interviewed caretakers whose children had recovered from convulsion episodes; if a child was still ill, they advised the caretaker to seek treatment from a health facility. The first author conducted a quality check for the EMIC; she revisited 15% of all households to assess the validity of the responses and all responses corresponded what had been earlier collected by the field staff. Households were geo-located and the information was linked with the data from a previous study [25] in order to calculate average distances from households to health facilities and from households to ADDOs.

### Data entry and analysis

STATA 10.0 was used to analyze quantitative information while MaxQDA [26] was applied for content analysis of illness narratives from the EMIC. The analysis followed the plan for the first convulsion study [14] with exception that the current study concentrated on only convulsion cases and not mild malaria. Differences in proportion between the 2004/06 and 2007/08 studies were compared using the two sample- binomial tests for proportions. Three outcome variables were defined to include 1) ‘Timely health facility use’ (HF use) 2) ‘Timely health facility and anti-malarial use’ (HF AM) and ‘Timely anti-malarial not from health facility’ (AM not HF). Multivariate logistic regression model was applied for the correlation of covariates with outcome variables. Distance calculations were carried out with ArcMap Version 9.1(ESRI Inc).

### Ethical review

This paper was published with the permission of Dr. Mwele Malecela, Director General, National Institute for Medical Research. Ethical Clearance of the ACCESS Programme proposal was granted by the National Institute for Medical Research of the United Republic of Tanzania (NIMR/HQ/R.8a/Vol.IX/236, September 16, 2003)

## Results

### Sample characteristics

During the second round of interviews, we found fewer cases of *degedege* (88 in 2007/08 as compared to 135 in 2004/06) although the seasonal coverage and the duration were nearly the same (17 and 15 months, respectively). The samples include 6 and 5 children from the fever studies [3,4] respectively in the 2004/6 and 2007/8 studies, whose illness had been identified as *degedege* by their caretakers. The samples were similar in terms of demographic characteristics (see Table 1): most of the interviewed caregivers were the mother of the sick child, married and depended on agriculture for their livelihood. However, there was a significant increase in households reporting uncertain or irregular income from 58/135 (43% 95%CI = 34% to 51%) in 2004/06 to 64//88 (73% 95%CI = 63% to 82%) in 2007/08 (p < 0.001). More caretakers stayed in the village at the onset of the illness (83/88 94% 95%CI = 89% to 98%) in 2007/08 compared to 2004/06 (107/135 79% 95%CI = 72% to 85%). In the second round of interviews, the average distance from the home to a health facility was 3.7 kilometers and 1.5 kilometers to a drug shop.

**Table 1:** Similarities and differences in the two study samples

	First study 2004/06 N = 135*	Second study 2007/08 N = 88*
	n (%)	n (%)
**Relationship to the Child:**		
Mother	116 (85.9%)	72 (81.8%)
Father	14 (10.4%)	10 (11.4%)
Grandmother	1.0 (0.7%)	4(4.5%)
Other	4.0 (3.0%)	2 (2.3%)
**Marital status:**		
Never married	13 (9.6%)	6 (6.8%)
Married	112 (83%)	72 (82.0%)
Separated, divorced	8 (5.9%)	5 (5.6%)
Widowed	1(0.7%)	4(4.5%)
Not specified	1(0.8%)	1(1.1%)
**Income:**		
regular and dependable	54 (40.0%)	21(24%)
Possibly	23 (17%)	3(3.4%)
Uncertain/irregular	58(43.0%)	64(72.6%)
**Occupation:**		
Farmer	128(94.8%)	85(96.7%)
Trade/Business	5(3.7%)	1(1.1%)
Laborer	2(1.5%)	1 (1.1%)
Teacher	0(0.0%)	1(1.1%)
**Location at time of Illness recognition:**		
Main village of residence	107(79.3%)	83(94.2%)
Temporary shelter in rice field *(shamba).*	28(20.7%)	5(5.8%)

### Degedege symptoms

**Figure 1:**
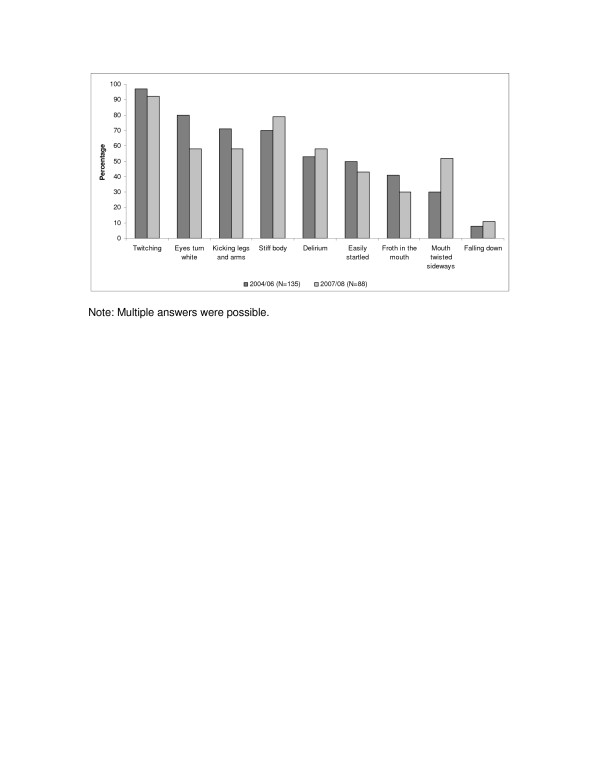
**Reported symptoms.** Note: Multiple answers were possible.

The experience of convulsions articulated in terms of five lead symptoms were nearly the same in both studies: “twitching” (*kustuka*), “eyes turn white” (*macho yanakuwa meupe*), “kicking of legs and arms” (*kurusha mikono na miguu*), “stiff body” (*mwili kukakamaa*) and “delirium” (*kuweweseka*) (see Figure 1).

The illness narratives showed that the percentage of caregivers who associated *degedege* with severe malaria was already high in the 2004/2006 study and rose, although not significantly, from 92/135 (68% 95CI = 60% to 76%) in 2004/2006 to 66/88 (75%, 95CI = 66% to 84%) in 2007/2008 (p = 0.261) (not shown in Table). Most caregivers (86/135 64% 95% = CI 55% to 72%) in 2004/06 and in 2007/08 (53/88 60% 95%CI = 50% to 70%) reported that the *degedege* episode started all of a sudden, without prior symptoms of an illness. As two women reported:

"“I woke up at 2 a.m. to find my child kicking her legs and arms. She had also high fever, and she was twitching” (mother aged 28 from Mbingu village, 2004/06)."

"“In the morning suddenly my child’s body was very hot. He started stretching his legs and arms, froth started coming out his mouth, his eyes were rolling. I carried him and rushed to the health facility” (mother aged 24 from Iragua village, 2007/08)."

### Perceived causes

**Figure 2:**
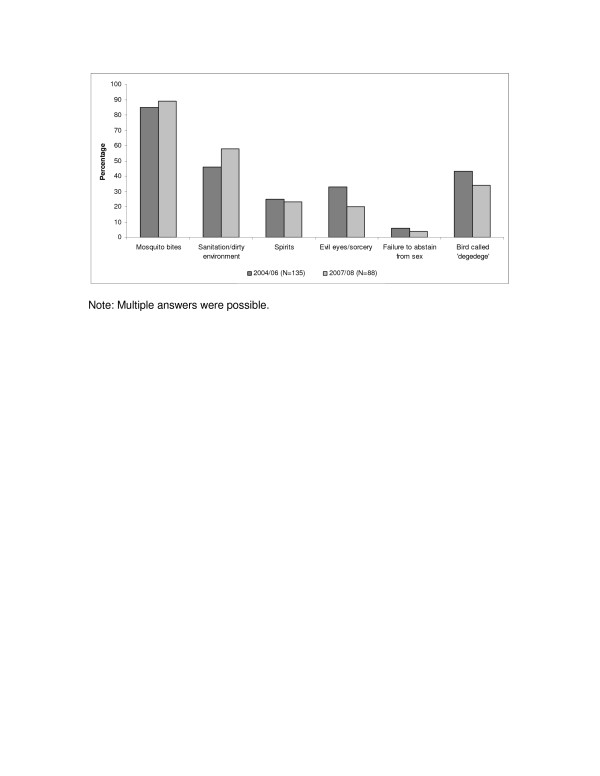
**Perceived causes.** Note: Multiple answers were possible.

Already in the first interview round, the percentage of caregivers who mentioned mosquito bites as a cause of *degedege* was high (115/135 85% 95CI = 79% to 91%); and the percentage has further increased though not significantly (78/88 89% 95CI = 84% to 96%) in 2007/2008 (p = 0.27) (Figure 2).

A slightly higher but insignificant percentage of study participants saw a causal link between “sanitation/dirty environment” and convulsions (62/135 46% 95% CI 37% to 54%) in 2004/06 compared to 2007/08 (51/88 58% 95% CI 48% to 68%) (p = 0.07). The qualitative data show that people consider toilets as well as grass, bushes and water pools around the house as breeding sites for mosquitoes causing *degedege*.

"“People are not used to keep the environment around houses clean. You see now, the grass around the houses is not cleared and there are many water pools. This attracts mosquitoes to breed” (mother aged 28 from Iragua village, 2007/08)."

At the same time, we see a small but consistent decrease in the percentage of “traditional” understandings of causes which have often been associated with *degedege*, such as the ‘insect *degedege*’ from 58/135 (43% 95%CI = 35% to 51%) to 30/88 (34% 95%CI =24% to 44%) (p = 0.18) “evil eye and sorcery” (*macho mabaya na uchawi*) from 45/135 (33% 95%CI = 25% to 40%) to 18/88 (20% 95%CI = 11% to 28%) (p = 0.03), “spirits” (*mapepo*) from 34/135 (25% 95%CI = 17% to 32%) to 20/88 (23% 95%CI = 14% to 32%) (p = 0.73), or “failure to abstain from sex” (*mzazi kushindwa kuacha vitendo vya ngono*) from 8/135 (6% 95%CI = 1% to 10%) to 4/88 (4% 95%CI = <0.1% to 8%) (p = 0.51).

These findings match with a significant increase in caregivers’ awareness (not shown in the table) that convulsions can be prevented with insecticide treated bed nets from 46/135 (34% 95%CI = 26% to 41%) in 2004/06 to 70/88 (80% 95%CI = 71% to 88%) (p < 0.001) in 2007/08 and environmental measures from 40/135 (30% 95%CI = 22% to 37%) in 2004/06 to 53/88 (60% 95%CI = 49% to 70%) (p < 0.001) in 2007/08.

"“Yes…to be honest majority of villagers now know that mosquito treated nets can prevent them from malaria and convulsion, we now have bed nets that have been treated for so many years and at least we can prevent ourselves from malaria” (mother aged 24 from Igota village, 2007/08)."

### Treatment seeking

**Figure 3:**
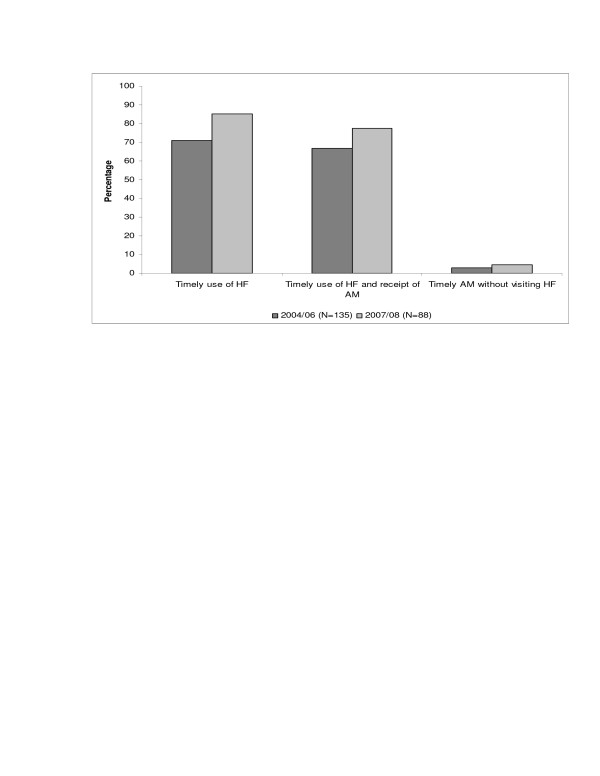
Treatment seeking.

In the second round of interviews, more caregivers brought children with convulsions to a health facility (see Figure 3) within 48 hours from the onset of symptoms 75/88 (85% 95%CI = 77% to 92%) compared to 96/135 (71% 95%CI = 63% to 78%) (p = 0.02) in 2004/06. There was also an increase, although not significant, in the number of those children who received an anti-malarial 68/88 (77% 95%CI = 68% to 85) compared to 90/135 (67% 95%CI = 59% to 74%) (p = 0.10) in 2004/2006. The percentage of caregivers who bought medicines from drug stores without prior visit to a health facility remained consistently low 4/135 (3% 95%CI = <0.1% to 5%) in 2004/06 and 4/88(4% 95%CI = <0.1% to 8%) (p = 0.69) in 2007/08 even though ADDO provide anticonvulsive medicine.

Caregivers explained their choice of health facilities as follows

"“Where else could I take my child for treatment? I carried her and went straight to the dispensary; this is where she could be treated” (mother aged 27 from Kivukoni village, 2007/08)."

"“After he [the child] started twitching the legs, I knew it was degedege because his body was also very hot. What I did is to rush him to the health facility because there are professionals who could treat him” (mother aged 26 from Lupiro village, 2007/08)."

Mothers evaluate the treatment of their children

"“It started with high fever then twitching, I knew this was degedege, I rushed my child to the dispensary….I believed she would get proper treatment there….After a week the child was back to normal health”(Mother aged 24 from Igota village, 2007/08)"

"“Yes she got treatment at Lupiro health centre…we were admitted for four days, they also gave us some tablets to take home….after about six days Mkeli (the child) was healthy again” (Mother aged 22 from Lupiro village, 2007/08)"

### Symptoms, causes and treatment outcome

We further examined whether reported symptoms and/or perceived causes predict treatment outcomes (see Table 2). All covariates were adjusted for potential confounders (i.e. age, sex, socio-economic status, marital status, health facility availability in the village and occupation) and only significant associated variables are presented. With regards to the treatment outcome “timely health facility use”, in the first round of interviews [14] we found a significant association with the reported symptoms “hot abdomen” (Estimate = 0.79) (p = 0.02) and “difficult breathing” (Estimate 0.87) (p = 0.04). However, neither these two nor any other reported symptoms turned out to be significantly correlated with “timely health facility” use in 2007/08. The main perceived cause which showed a significant correlation with “timely health facility use” in the first study (Estimate 1.54) (p < 0.001) also did so in the second study (Estimate 1.43) (p = 0.04): “sanitation/dirty environment”. In the first study, the more “traditional” understanding of what causes convulsions such as “spirits” (Estimate-1.72) (p = 0.03) and “a failure to abstain from sex” (Estimate −2.84) (p = 0.03) tended to keep caregivers from prompt help seeking at the health facility. A similar association was not found in the second study.

**Table 2:** Reported symptoms, perceived causes and treatment outcomes for convulsion

	2004-2006						2007-2008					
Percentage of respondents who mentioned the outcome	HF Use 71%		HF AM^2^ 67%		AM not HF^3^ 3%		HF Use 85%		HF AM 77%		AM not HF 5%	
	Estimate	p	Estimate	p	Estimate	p	Estimate	p	Estimate	p	Estimate	p
Reported Symptoms												
No interest to play	1.16	0.12	2.52	<0.001								
Hot abdomen	0.79	0.02	0.56	0.05								
Difficult breathing	0.87	0.04	1.55	<0.001								
*Degedege* symptoms^4^					−2.65	0.04					15.16	<0.001
Perceived Causes												
Constitution/blood weakness	3.03	0.01	2.95	<0.001								
Sanitation/dirty environment	1.54	<0.001					1.43	0.04	1.39	0.03		
Bird/insect called *degedge*			−0.60	<0.001								
Spirits	−1.72	0.03										

Note: Model fitness based on the likelihood ratio (all models with p < 0.0001).

1 Model outcome: health facility immediate use (HF use) (same day or next day).

2 Model outcome: health facility and anti-malarial immediate use (HF AM) (same day or next day).

3 Model outcome: anti-malarial immediate use not from the health facility (AM not HF) (same day or next day): the definition of this category differs from the definition used in Table four of the baseline study [12] because we regrouped this category to include only AM from the drug shop without prior visit to health facility.

4 Grouped *degedege* symptoms include responses mentioning at least two of the following symptoms; twitching, body becomes stiff, delirium, eyes turn white, kicking of legs and arms, froth in the mouth, mouth twisted sideways, falling down, easily startled.

The second treatment outcome, “timely health facility and anti-malarial use”, was associated with the symptoms “no interest to play” (Estimate 2.52) (p < 0.001) and “difficult breathing” (Estimate1.55) (p < 0.001) in the first study. However, it was not associated with any symptom in the 2007/08 study. With regard to the perceived causes, one showed a significant association in the first study, namely “constitution/blood weakness” (Estimate 2.95) (p < 0.001) while “sanitation/dirty environment” (Estimate 1.39) (p = 0.03) did so in the second study. Only in 2004/06, caretakers who reported the ‘bird *degedege’* as the cause for convulsion were less likely to use health facility timely and receive anti-malarials (Estimate −0.60) (p < 0.001). Also, the findings do not demonstrate any difference in treatment seeking patterns between caregivers who recognized the child’s illness at home as compared to those who recognized the illness while they stayed in their farms (*shamba*) (data not shown).

The percentages for the last treatment outcome, ‘timely anti-malarial not from the health facility’, were too low to be included into a more detailed analysis.

### Affordability and traditional medicine

The proportion of caregivers who delayed attending a health facility until the third day remained similar in 2007/08 with 9/88 (10% 95%CI = 3% to 16%) compared to 11/135 (8% 95%CI = 3% to 12%) (p = 0.61) in 2004/06, but the percentage of those who did not use a health facility at all dropped remarkably, from 28/135 (21% 95%CI = 14% to 27%) in 2004/06 (these include 3% who received anti-malarials from another source) to 4/88 (5% 95%CI = <0.1% to 9%) (p < 0.001) in 2007/08. A closer look at the illness narratives shows that affordability and/or acceptability dimensions of access played a role in delaying treatment:

"“We gave the child manunganunga medicine (traditional medicine) to decrease the speed of that mdudu degedege (the degedege insect), we sponged him with the medicine and also we gave him some medicine to drink. We could not go straight to the health facility as we did not have money. On the third day, we were able to reach the facility with cash” (mother aged 30 from Idete village, 2007/08)."

"“When you take a convulsed child to a health facility and he receives an injection, he experiences a lot of pain. So we decided to sponge him with traditional medicine first to calm down the degedege and went to the hospital on the third day” (mother aged 21 from Iragua village, 2007/08)."

"“I went to the traditional healer, this is where degedege is treated. The healer sponged the child with some medicine. Then the convulsions calmed down. He also used a piece of cloth filled with elephant dung and tied it around the child’s wrist: We went home, and the child was healed. We didn’t take her to the health facility” (Mother aged 28 from Idete village, 2007/08)."

"“We have been to the hospital more than three times for convulsions this year. I think their medicines are not working. This time, I chose to go straight to the traditional healer, and my child is doing fine now” (Mother aged 18 from Idunda village, 2007/08)."

The cases documented here show that opting for traditional treatment is one thing, and the reasons for doing so is another: In the first case, the caregivers had to mobilize money before they could obtain health facility treatment for their child, in the second case, they first recurred to local treatment because they wanted to spare the child from a painful injection. The third mother did not see a reason to bring the child to the clinic because the symptoms disappeared after “traditional” treatment. The fourth mother decided based on previous experience that her child’s convulsions could not be cured with biomedicine.

### Gender and treatment decision making

In both interview rounds, most caregivers 131/135 (97% 95% CI = 94% to 99%) and 72/88(82% 95%CI = 74% to 90%), respectively, reported that they did not consult anyone for advice on where to take the child. They said they knew themselves what illness their child suffered 68/135(50% 95% CI = 41% to 58%) and 66/88(80% 95%CI = 71% to 88%). None of the mothers in either study mentioned that they had consulted the husband, and only few 7/135(5% 95CI = 1% to 8%) and 8/88(9% 95%CI = 3% to 15%) (p = 0.24) sought assistance from an elderly woman (*bibi*).

## Discussion

Our findings demonstrate that the match between local and biomedical understandings of convulsions was already high in 2004/06 [14] and we noticed significant changes in the second round of interviews (2007/08) specifically on; 1) increase in percentage for those who reported mosquito nets as measure to prevent convulsion, from 46/135 (34% 95%CI = 26% to 41%) in 2004/06 to 70/88 (80% 95%CI = 71% to 88%) (p < 0.001) in 2007/08, 2) environmental measures from 40/135 (30% 95%CI = 22% to 37%) in 2004/06 to 53/88 (60% 95%CI = 49% to 70%) (p < 0.001) in 2007/082), 3) decrease in percentage of caregivers who associated “evil eye and sorcery” (*macho mabaya na uchawi*) and convulsion from 45/135 (33% 95%CI = 25% to 40%) to 18/88 (20% 95%CI = 11% to 28%) (p = 0.03), 4) a 14 percentage point increase in prompt use of a health facility for children with convulsion, 75/88 (85% 95%CI = 77% to 92%) compared to 96/135 (71% 95%CI = 63% to 78%) (p = 0.02) in 2004/06 and 5) a 16 percentage point decrease for those who did not use health facility at all, from 28/135 (21% 95%CI = 14% to 27%) in 2004/06 to 4/88 (5% 95%CI = <0.1% to 9%) (p < 0.001) in 2007/08.

Clearly, a change in the social acceptability of malaria prevention and biomedical treatment of convulsions has occurred since the late 1990s, when medical anthropologists carried out the first studies in this same research area [27,28]. At that time, the health facility was a second or third choice after convulsions were calmed down by locally known practices and/or after such treatment failed and the child’s condition worsened. This change can to a large extent be attributed to the three initiatives which explicitly aimed at increasing the match between local and biomedical understanding and treatment of malaria: the KINET project (1996–2000), IMCI implementation (since 2002) and the ACCESS project (since 2004). All three initiatives took malaria-related values and understandings that existed in the society into account, and this is a major step towards improving the match between local and external knowledge [28]. Even though a proper case control study was not carried out, it seems reasonable to associate the increased social acceptability which manifests itself in improved practices to such interventions. Higher percentages of caregivers’ positive behaviour towards the understanding and treatment of convulsion that is already noticed in our first study [14] is more likely to be the effect of the KINET and IMCI interventions as no other studies (apart from the ACCESS study [14]) were conducted in the area to explore changes in community understanding and treatment practices for convulsions after the two interventions were ended. Other studies provide additional evidence for the positive impact of social marketing and other communication campaigns on the social acceptability of malaria prevention and treatment in Tanzania and Africa, for instance with regard to IPTi interventions [29] and home-based management of malaria [30,31]. Despite ADDOs being closer to their homes than health facilities, caregivers by-passed these outlets and sought treatment at health facilities. This finding is contrary to what is reported by Rutebemberwa et al., (2009) whereby caregivers living within long distances to providers were more likely to delay seeking treatment [32]. It should be noted that ADDO dispensers had received training on IMCI. In cases of severe malaria with convulsion, immediate referral to health facility is recommended. It is also likely that caregivers had received information on where to treat a seriously ill child through their prior experiences with ADDOs.

The increased social acceptability of biomedical malaria prevention and treatment may even have contributed to the decline of *degedege* incidences which was noted in the two interview rounds within the ACCESS program (from 131 in 2004/06 to 88 in 2007/08) and which has also been reported in the study area [33]. This is consistent with other studies in the same area which have documented a sharp decrease in malaria transmission [22,34].Interestingly, the findings presented here also indicate perceived efficacy of modern treatment for convulsion among caregivers.

While a number of studies in Africa have indicated consultations of respectable community members including old women for advice in cases of convulsion [35,36], our study attested caregivers’ independence in recognizing convulsion symptoms and seeking appropriate care. This is more surprising since the majority of caregivers in our samples were women whose autonomy in health decision making is commonly limited in African societies [15,37,38]. Our findings may reflect the fact that biomedical malaria prevention and treatment has become socially acceptable, also for severe fever cases with convulsions, so that mothers do not need to ask for consultation or permission any more.

Most of the literature on malaria and convulsions in Tanzania and the studies reviewed by William and Jones [9] did not explicit refer to the concept of acceptability. Our study suggests that the concept of social acceptability can help to link studies on local vs. biomedical understandings of illness with broader debates on access to prevention and treatment technologies and associated services. Further studies on social acceptability should also look at health practitioners and health policies and investigate whether they take local illness understandings into account. They should also explore further meanings of the concept of social acceptability, for instance questions concerning the match of ethics and morality in interactions between health workers and caregivers.

## Conclusions

‘Social acceptability’ as an important access dimension to health care seems more relevant in studying illnesses that are perceived by communities as not belonging to the biomedical field i.e. convulsion, with this regard, the match between local and biomedical understandings of health problems is central in understanding the concept of acceptability and especially in trans-cultural societies. Study results indicate some positive changes in community understanding and treatment practices for childhood convulsion from the baseline study; however our findings can not be generalized because of the small sample size involved and the influence of long-term health intervention activities in the study area. To improve social acceptability of treatment, it is important to take up existing local words and treatment practices into interventions and involve communities at all levels of the interventions. Moreover the quality of care and efficacy of treatment on the supply side is of relevance to ensure community acceptability of health services.

## Competing interests

The authors declare that they have no competing interests.

## Authors’ contributions

AD was involved in the design and implementation of the study, field work, data management, analysis, interpretation and writing of the manuscript. AS, BO, CL, HM conceived the program and its components and provided technical support and supervision. MW was involved in the design and analysis of the EMIC. BO was involved in the conception and design of the study, data analysis, interpretation and writing of this paper. MH and SA were involved in the design and implementation of the studies and IM contributed to data collection and discussion of the findings. CM was responsible for the development and implementation of the interventions. All authors read and approved the final manuscript.

## Pre-publication history

The pre-publication history for this paper can be accessed here:

http://www.biomedcentral.com/1472-6963/12/113/prepub
